# Interpretable RR Interval-Based ECG Rhythm Abnormality Detection: Cross-Dataset Evaluation, Feature Explainability, and Feature-Level Sensitivity Analysis

**DOI:** 10.3390/diagnostics16213561

**Published:** 2026-10-29

**Authors:** Akanksha Gupta, Manav Bhatnagar

**Affiliations:** 1 Bharti School of Telecommunication Technology & Management, Indian Institute of Technology Delhi, Hauz Khas, New Delhi 110016, India; 2 Department of Electrical Engineering, Indian Institute of Technology Delhi, Hauz Khas, New Delhi 110016, India; manav@ee.iitd.ac.in

**Keywords:** electrocardiogram, abnormal rhythm variability, heart rate variability, feature ablation analysis, cross-dataset evaluation

## Abstract

**Background/Objectives:** Interpretable ECG rhythm classification remains challenging because of variability in recording conditions, patient characteristics, and annotation practices across datasets. Although deep learning models can achieve high classification performance, their limited interpretability and sensitivity to domain shift can restrict their applicability in real-world clinical settings. This study investigates an interpretable and computationally efficient framework for ECG rhythm abnormality detection using statistical descriptors of RR interval variability. **Methods:** Mean RR, SDNN, RMSSD, pNN50, and heart rate features were extracted from ECG recordings to characterize beat-to-beat temporal variability. A Random Forest classifier was employed for binary rhythm classification using the PhysioNet/CinC 2017 dataset, followed by cross-dataset testing on the ECG-SDS dataset, which consists of synthetic ECG recordings. Same-dataset evaluation was additionally performed on ECG-SDS to establish a same-dataset performance benchmark. Feature ablation and feature-level sensitivity analyses using controlled Gaussian perturbations were conducted to examine feature contribution and classifier sensitivity to perturbations in the extracted feature representation. Multi-class experiments were also performed by incorporating atrial fibrillation and sinus tachy-brady rhythms. **Results:** In binary cross-dataset evaluation, the proposed framework achieved an accuracy of 78.6%, abnormal rhythm recall of 74.2%, and ROC-AUC of 0.836 on ECG-SDS. The same-dataset ECG-SDS evaluation achieved an accuracy of 93% and ROC-AUC of 0.89. Ten-fold stratified cross-validation yielded a mean accuracy of 92.20% ± 2.63% and mean AUC of 0.8880 ± 0.0477. Feature ablation showed only modest performance changes when individual descriptors were removed. The exploratory same-dataset multi-class evaluation on ECG-SDS achieved an accuracy of 91.67%. **Conclusions:** The results demonstrate that compact RR interval statistical descriptors can provide an interpretable and computationally efficient representation for ECG rhythm classification. The reduction in performance during cross-dataset testing may reflect dataset shift and differences in model configuration. These cross-dataset results should be interpreted as a technical assessment of transferability rather than as validation in an independent clinical population. Further validation using independent real-world clinical ECG datasets is required before conclusions regarding clinical generalizability or screening applications can be drawn.

## 1. Introduction

Electrocardiography (ECG) is one of the most widely used non-invasive diagnostic techniques for monitoring cardiac activity and detecting rhythm abnormalities [1]. Accurate ECG rhythm classification is essential for the early diagnosis of cardiovascular diseases, continuous patient monitoring, and large-scale clinical screening. With the rapid growth of digitally recorded ECG data, machine learning (ML) has become an important tool for automated ECG analysis. Existing approaches range from conventional methods based on handcrafted features to advanced deep learning models [2,3,4].

Recent deep learning methods have reported impressive performance for ECG rhythm classification [5,6,7]. Despite these advances, most deep learning models rely on large annotated datasets, high computational resources, and extensive hyperparameter optimization. In addition, their limited interpretability makes it difficult for clinicians to understand the reasoning behind model predictions, which remains a major challenge for real-world clinical adoption [8,9].

To address the need for more interpretable models, many researchers have explored heart rate variability (HRV) analysis using RR intervals. Statistical RR interval features such as mean RR, SDNN, RMSSD, pNN50, and heart rate capture important physiological characteristics of cardiac rhythm while remaining computationally efficient and easy to interpret [10,11,12]. Previous studies have shown that combining these HRV features with traditional machine learning classifiers can provide reliable performance for ECG rhythm classification [13,14].

Although considerable progress has been made, most ECG classification studies evaluate their models using training and testing data from the same dataset [15]. Such evaluation protocols often produce optimistic results because they do not account for variations in acquisition devices, patient populations, signal quality, rhythm distributions, and annotation standards across different datasets [16]. As a result, models that perform well on one dataset frequently experience reduced performance when applied to independent datasets. Recent reviews therefore highlight cross-dataset validation as an important step toward developing ECG analysis systems that are reliable in real clinical settings [17]. However, only a limited number of studies have simultaneously examined RR interval interpretability, cross-dataset generalization, feature explainability, and robustness within a single experimental framework.

Motivated by these limitations, this work investigates whether a compact and physiologically meaningful set of RR interval features can support ECG rhythm classification across datasets with differing characteristics. Rather than proposing a new machine learning algorithm or introducing additional handcrafted features, this study focuses on a comprehensive evaluation of RR interval-based ECG rhythm analysis through cross-dataset evaluation, feature-level explainability, feature-level sensitivity analysis, and classifier comparison. The primary emphasis is on binary rhythm abnormality classification, while an exploratory same-dataset multi-class experiment is included to examine classification performance across the rhythm categories represented in the synthetic ECG-SDS dataset.

The proposed approach extracts statistical RR interval descriptors from single-lead ECG recordings and employs a Random Forest classifier for rhythm classification. The model is trained using the PhysioNet/CinC 2017 dataset [18] and evaluated on the independent ECG-SDS dataset [19], providing a realistic assessment under heterogeneous ECG acquisition conditions. To further investigate the effect of dataset shift, additional same-dataset experiments are performed. The evaluation is complemented by feature ablation studies, feature importance analysis, comparisons with multiple machine learning classifiers, feature-level sensitivity analysis using controlled Gaussian perturbations, and exploratory multi-class classification experiments.

The main contributions of this work are summarized as follows:

- An interpretable framework for RR interval-based ECG rhythm classification is presented through cross-dataset evaluation, feature explainability, feature-level sensitivity analysis, and classifier comparison.
- Binary classification experiments are conducted under same-dataset and cross-dataset settings, together with an exploratory same-dataset multi-class analysis, to assess classification performance and cross-dataset transferability between the evaluated datasets.
- Feature ablation and Random Forest feature importance analyses are performed to evaluate the contribution and interpretability of individual RR interval descriptors.
- A feature-level sensitivity analysis is performed by applying controlled Gaussian perturbations to standardized extracted features to examine the stability of classifier performance under perturbations in the feature representation.
- Comparative experiments with multiple machine learning classifiers and comprehensive performance metrics are used to characterize the performance of the proposed RR interval-based classification framework.

## 2. Dataset Used

Two independent ECG datasets were used in this study to evaluate both classification performance and model generalization across different data sources. The PhysioNet/Computing in Cardiology (CinC) 2017 dataset [18] was used for model development and training, while the ECG-SDS dataset [20] was used in two separate experimental settings: intra-dataset evaluation and cross-dataset testing for technical evaluation of transferability. Cross-dataset testing provides a technical assessment of model transferability under dataset shift beyond conventional same-dataset evaluation.

The PhysioNet/CinC 2017 dataset is a publicly available collection of single-lead ECG recordings collected from a variety of clinical and ambulatory settings. The recordings range from approximately 9 to 60 s in duration and were originally sampled at around 300 Hz. To ensure consistency during RR interval extraction, all recordings were processed using the original sampling frequency of 300 Hz. The original REFERENCE.csv annotation file contained 8528 ECG recordings, comprising 5050 Normal (N), 738 Atrial Fibrillation (A), 2456 Other (O), and 284 noisy (∼) recordings. Following preprocessing and successful RR interval feature extraction, 8523 recordings were retained, while 5 recordings were excluded because valid features could not be obtained. Specifically, the retained recordings comprised 5049 N, 738 A, 2453 O, and 283 ∼ recordings, with 1, 0, 3, and 1 recordings excluded from the respective categories. For the original binary cross-dataset analysis, the A, O, and ∼ categories were grouped into a single abnormal class, resulting in 5049 normal and 3474 abnormal retained recordings.

To examine the potential effect of including technically noisy recordings in the abnormal class, an additional sensitivity analysis was performed after excluding all 283 retained ∼ recordings from model development. The resulting source dataset contained 8240 recordings, comprising 5049 Normal, 738 Atrial Fibrillation, and 2453 Other recordings; under the binary formulation, this corresponded to 5049 normal and 3191 abnormal recordings.

The ECG-SDS dataset is a commercially licensed synthetic ECG database obtained through QInsight Lab for academic research purposes [20]. Owing to licensing restrictions, the dataset cannot be publicly redistributed by the authors. Researchers interested in reproducing the experiments can obtain the ECG-SDS dataset directly from QInsight Lab through its official dataset access page, subject to the provider’s current purchase and licensing conditions. The dataset is available at https://qinsight.gumroad.com/l/kupvpv (accessed on 20 Febuary 2026). It contains 1500 synthetic single-lead Lead-II ECG recordings sampled at 250 Hz, with each recording having a fixed duration of 10 s. The recordings are annotated as normal sinus rhythm, atrial fibrillation, or sinus tachy–brady rhythm.

Since the two datasets follow different rhythm taxonomies, their class labels were not considered clinically equivalent on a one-to-one basis. For the ECG-SDS same-dataset experiments, the three classes were defined as Normal, Sinus Tachy–Brady, and AFib based on the dataset-specific annotations. In comparison, the PhysioNet/CinC 2017 dataset includes the categories Normal (N), Atrial Fibrillation (A), Other (O), and noisy (∼). The ‘Other’ category represents a heterogeneous annotation in the PhysioNet/CinC 2017 dataset and should therefore not be treated as equivalent to the specifically defined Sinus Tachy–Brady class in ECG-SDS.

For the original binary cross-dataset evaluation, the PhysioNet/CinC 2017 categories A, O, and ∼ were combined into a single abnormal class, while N represented the normal class. Because a technically noisy or uninterpretable recording does not necessarily indicate a cardiac rhythm abnormality, an additional sensitivity analysis excluded the 283 retained ∼ recordings from source model development. In this analysis, A and O constituted the abnormal source class, and N constituted the normal source class. In ECG-SDS, atrial fibrillation and sinus tachy–brady rhythm were combined into the abnormal class, while normal sinus rhythm represented the normal class. Because the dataset-specific rhythm taxonomies do not support a clinically meaningful one-to-one mapping of all multi-class labels, multi-class evaluation was restricted to the same-dataset ECG-SDS setting.

For the primary evaluation, the rhythm labels were reformulated as a binary classification problem. Normal sinus rhythm was treated as the normal class, whereas atrial fibrillation and sinus tachy–brady rhythm were combined into a single abnormal class. This screening-oriented formulation helps reduce annotation inconsistencies, differences in rhythm taxonomy between datasets, and class imbalance, making cross-dataset evaluation more reliable. After preprocessing, the ECG-SDS test set consisted of 1058 normal recordings and 442 abnormal recordings. Although ECG-SDS is not publicly available, it provides a synthetic dataset with signal characteristics, rhythm distributions, and annotation definitions that differ from those of PhysioNet/CinC 2017. Therefore, its use in the cross-dataset experiment provides a technical assessment of transferability under dataset shift rather than validation in an independent clinical population. To facilitate reproducibility, the preprocessing steps, RR interval feature extraction procedures, classifier settings, and evaluation protocols are described in detail. Researchers who do not have access to ECG-SDS can reproduce the proposed methodological pipeline using the publicly available PhysioNet/CinC 2017 dataset, whereas replication of the ECG-SDS experiments requires access to the dataset from the provider under the applicable licensing conditions.

In addition to the binary classification experiments, an exploratory multi-class evaluation was performed under the same-dataset ECG-SDS setting using normal sinus rhythm, atrial fibrillation, and sinus tachy–brady rhythm. Cross-dataset multi-class evaluation was not performed because the rhythm taxonomies of the two datasets do not provide compatible class definitions for a clinically meaningful one-to-one multi-class comparison.

Overall, the two datasets differ in signal characteristics, rhythm distributions, and annotation practices, providing a useful setting for the technical assessment of cross-dataset transferability. At the same time, both datasets contain single-lead ECG recordings, allowing the same RR interval feature extraction framework to be applied across the two data sources.

The ECG-SDS dataset was used in two distinct experimental settings. First, an intra-dataset evaluation was conducted using ECG-SDS data with a stratified 70:30 train–test split to assess the performance of the proposed approach under a controlled same-dataset setting. Second, ECG-SDS was used as the synthetic target dataset in the cross-dataset evaluation, where the classifier was fitted using the PhysioNet/CinC 2017 source dataset and subsequently evaluated on ECG-SDS. These two experimental settings are reported separately. Details of the cross-dataset model configuration and decision threshold analysis are provided in Section 3.6.

## 3. Methods

### 3.1. System Framework

**Figure 1:**
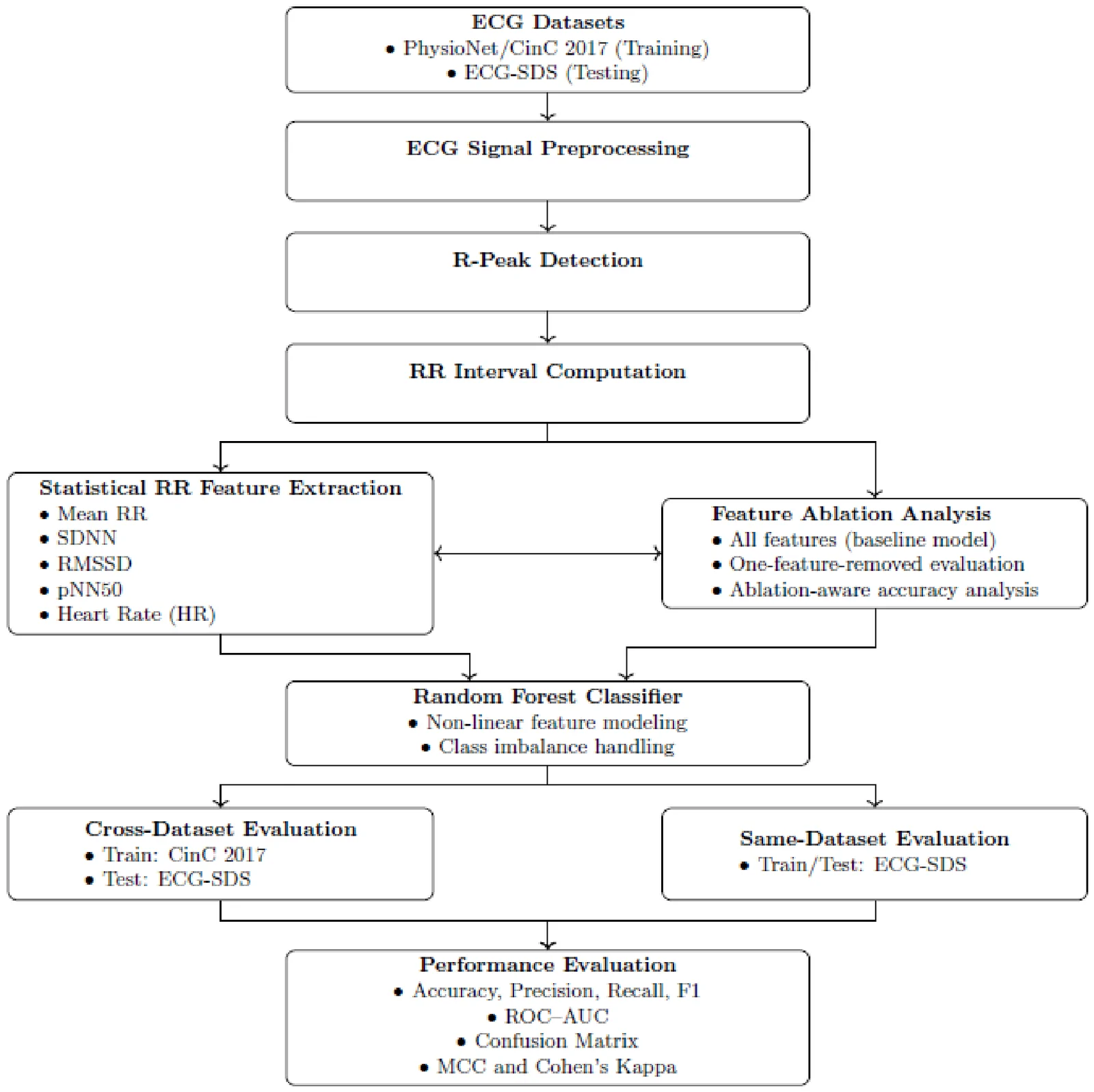
Workflow of the proposed RR interval-based ECG rhythm classification framework.

In this work, a unified pipeline for ECG rhythm classification based on RR interval variability analysis is developed. ECG recordings from the PhysioNet/CinC 2017 and ECG-SDS datasets were processed at their native sampling frequencies without explicit resampling, separate user-defined filtering, or amplitude normalization. The signals were supplied to the NeuroKit2 R-peak detection procedure using their corresponding sampling frequencies. R-peaks [21] were then detected from the ECG signals, and successive RR intervals were computed to capture temporal rhythm information. Statistical RR interval features were subsequently extracted and used for Random Forest-based classification. The performance of the proposed framework was evaluated in both same-dataset and cross-dataset settings using standard classification metrics. A schematic overview of the overall framework is illustrated in Figure 1.

### 3.2. Pre-Processing

ECG recordings from the two datasets differ in sampling frequency and recording duration. To preserve the native temporal characteristics of each dataset, the signals were processed at their original sampling frequencies rather than being resampled to a common frequency. Specifically, the PhysioNet/CinC 2017 recordings were processed at 300 Hz, whereas the ECG-SDS recordings were processed at 250 Hz.

No explicit resampling, separate Butterworth filtering, or user-defined amplitude normalization was applied to the ECG signals in the final RR feature extraction pipeline. Each ECG signal was supplied directly to the NeuroKit2 (v0.2.13) R-peak detection procedure (nk.ecg_peaks) together with its corresponding sampling frequency. Because no alternative detection method was specified in the implementation, nk.ecg_peaks used its default method=’neurokit’ setting. No additional manually specified amplitude, height, prominence, or minimum-distance thresholds were introduced for R-peak detection. This preprocessing description reflects the implementation used to generate the RR-derived feature matrix evaluated in the reported experiments.

### 3.3. R-Peak Detection and RR Interval Computation

**Figure 2:**
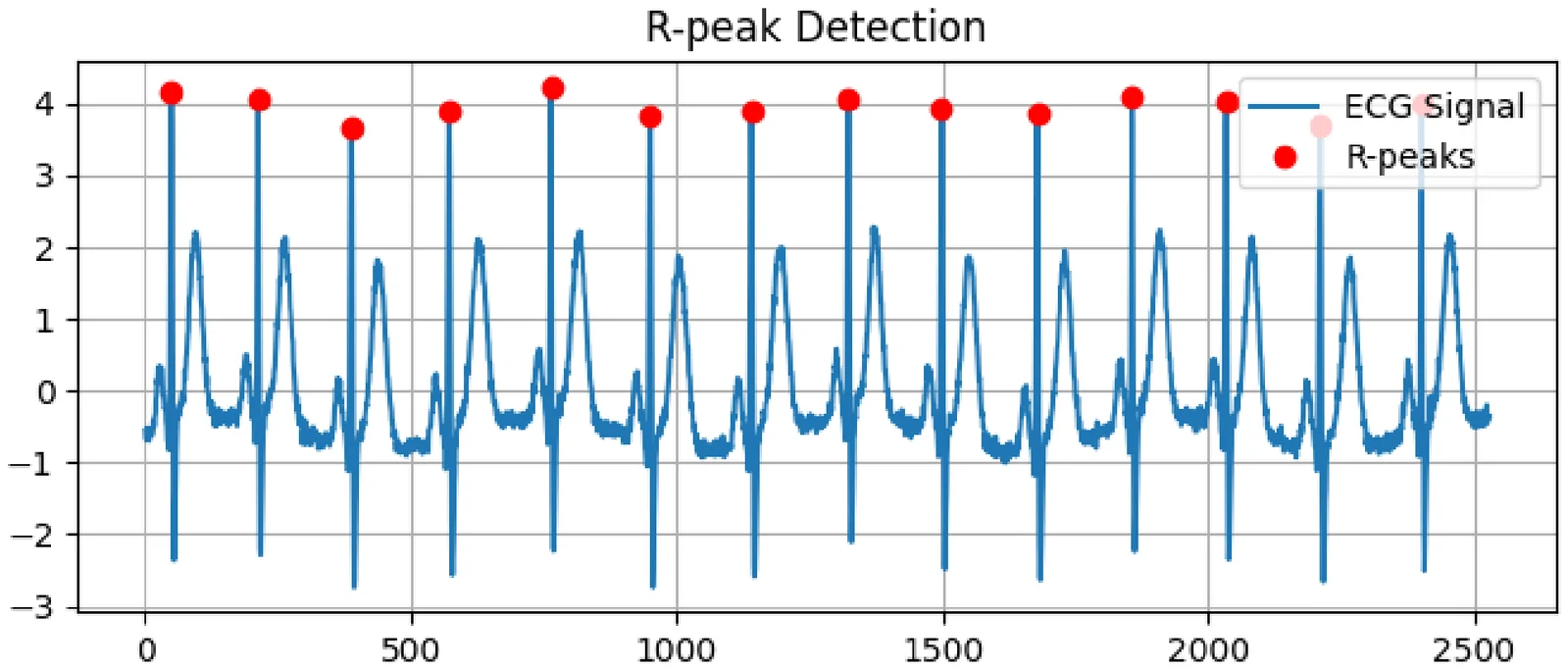
R-peak detection results showing the ECG signal and detected R-peaks marked as red circles.

**Figure 3:**
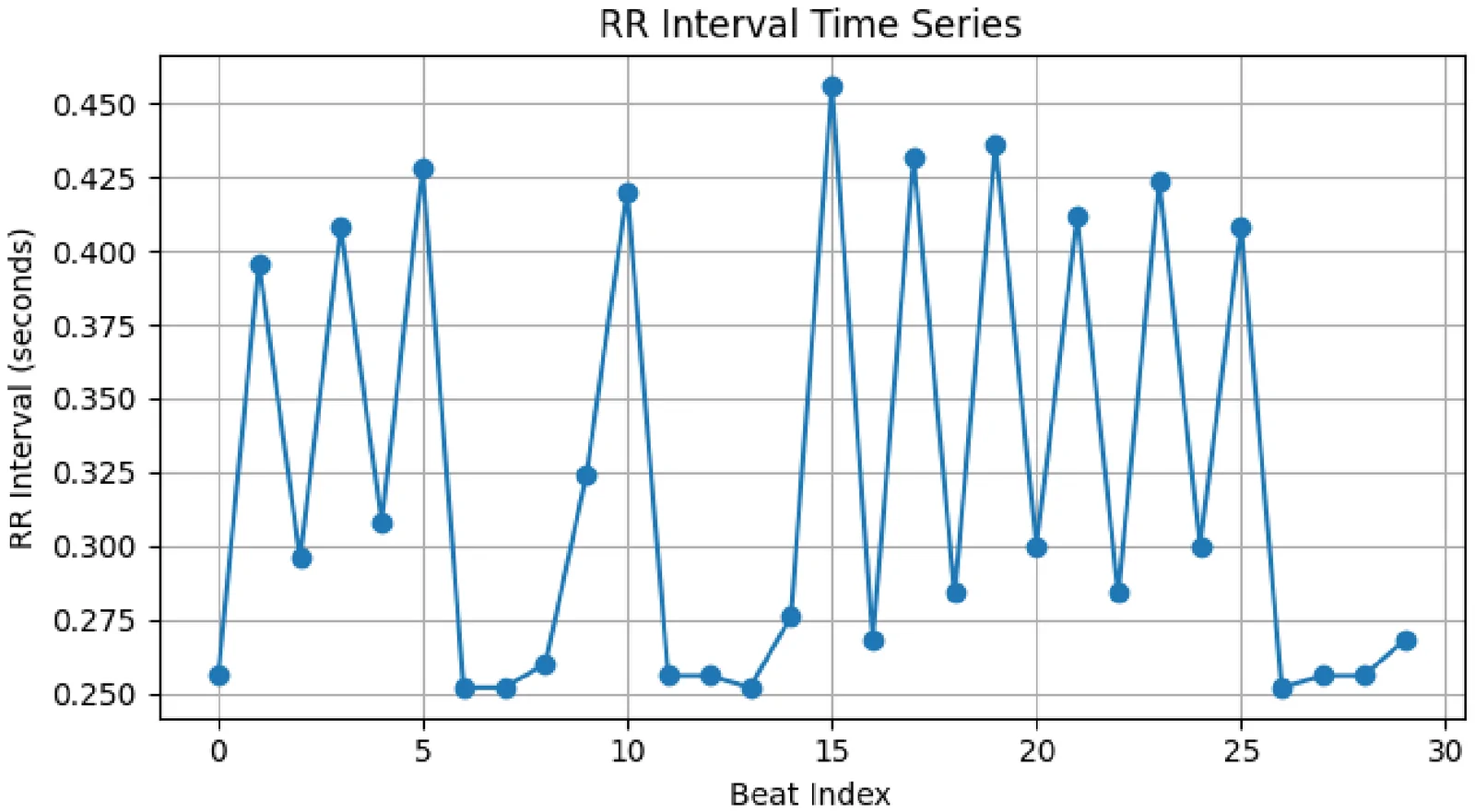
RR interval time series extracted from an ECG signal, illustrating beat-to-beat heart rhythm variability used for rhythm-level feature computation.

As illustrated in Figure 2, accurate R-peak detection is the first step in estimating inter-beat intervals. The detected R-peaks are used to generate the RR interval sequence, which captures beat-to-beat variations in cardiac rhythm, as illustrated in Figure 3, and serves as the basis for subsequent statistical feature extraction [22].

Reliable R-peak detection is essential because any error in heartbeat localization directly affects RR interval estimation and, consequently, heart rate variability analysis [23]. In this work, R-peaks were detected using the nk.ecg_peaks function from the NeuroKit2 library, with the sampling frequency of each recording supplied to the function. No additional manually specified amplitude, height, prominence, or minimum-distance thresholds were introduced in the implemented R-peak detection code. The original sampling frequencies of 300 Hz for the PhysioNet/CinC 2017 dataset and 250 Hz for the ECG-SDS dataset were used.

Following R-peak detection, the locations of consecutive R-peaks, denoted by sample indices $r_{i}$, were used to calculate successive RR intervals in seconds as

$$RR_{i}=\frac{r_{i+1}-r_{i}}{f_{s}},$$

where $f_{s}$ denotes the sampling frequency of the corresponding recording. All available RR intervals within each recording were used for feature calculation; no fixed-length selection or truncation to 30 RR intervals was applied. Recordings yielding fewer than three RR intervals were excluded from feature generation. Thus, at least three RR intervals, corresponding to at least four detected R-peaks, were required for feature calculation.

For the PhysioNet/CinC 2017 dataset, 8523 of 8528 recordings satisfied this criterion and were retained. Specifically, the retained class counts were 5049 Normal, 738 Atrial Fibrillation, 2453 Other, and 283 noisy recordings. Five recordings were excluded: 1 Normal, 0 Atrial Fibrillation, 3 Other, and 1 noisy recording. All 1500 ECG-SDS recordings yielded valid RR-derived feature vectors and were retained in the final analysis.

Among the 8523 eligible PhysioNet/CinC 2017 recordings, recording duration ranged from 9.05 to 60.95 s, with a mean of 32.50 s (SD: 10.89 s) and a median of 30.00 s (IQR: 30.00–30.00 s). The number of detected R-peaks ranged from 4 to 172, with a mean of 40.22 (SD: 17.12) and a median of 36 (IQR: 31–44). The number of available RR intervals ranged from 3 to 171, with a mean of 39.22 (SD: 17.12) and a median of 35 (IQR: 30–43). Only 6 eligible recordings contained fewer than 5 RR intervals, and 32 contained fewer than 10 RR intervals.

Mean RR, SDNN, RMSSD, pNN50, and heart rate were calculated directly from the RR intervals in seconds. The RR interval sequence was not normalized on a recording-by-recording basis before feature calculation. Mean RR was computed as the average RR interval, SDNN as the standard deviation of the RR intervals, RMSSD from successive RR interval differences, pNN50 as the proportion of successive RR interval differences exceeding 50 ms, and heart rate as 60/MeanRR. Thus, the physiological interpretation of the RR-derived features was preserved.

Beat-level reference annotations for R-peak locations were not available in the PhysioNet/CinC 2017 data used for the present feature-extraction pipeline. Therefore, reference-based sensitivity and positive predictivity of individual R-peak detections could not be directly quantified against an annotated beat-level reference. This limitation is acknowledged, and the implemented R-peak detection and RR interval computation procedure is reported to improve methodological transparency and reproducibility.

### 3.4. Statistical RR Interval Feature Extraction

After R-peak detection, a set of statistical features is extracted from the RR interval sequence to quantitatively characterise heart rhythm variability. Let the RR interval sequence be denoted $\left\{RR_{1},RR_{2},\ldots ,RR_{M}\right\}$, where each $RR_{i}$ represents the time difference between two consecutive R-peaks. These features capture both overall rhythm regularity and short-term beat-to-beat variability, which are clinically meaningful indicators of abnormal cardiac activity.

The mean RR interval (MeanRR), representing the average duration between successive heartbeats, is computed as

(1) $$\mathrm{MeanRR}=\frac{1}{M}\sum_{i=1}^{M}RR_{i}$$

Global heart rate variability is quantified using the standard deviation of RR intervals (SDNN), defined as

(2) $$\mathrm{SDNN}=\sqrt{\frac{1}{M-1}\sum_{i=1}^{M}{\left(RR_{i}-\mathrm{MeanRR}\right)}^{2}}$$

Short-term variability is captured by the root mean square of successive differences (RMSSD), which emphasizes rapid beat-to-beat changes:

(3) $$\mathrm{RMSSD}=\sqrt{\frac{1}{M-1}\sum_{i=1}^{M-1}{\left(RR_{i+1}-RR_{i}\right)}^{2}}$$

Another short-term variability measure is pNN50, defined as the percentage of successive RR interval pairs that differ by more than 50 ms:

(4) $$\mathrm{pNN}50=\frac{1}{M-1}\sum_{i=1}^{M-1}I\;\left(\left|RR_{i+1}-RR_{i}\right|>50\;\mathrm{ms}\right)\times 100$$

where I(·) denotes the indicator function.

Finally, heart rate (HR) is derived from the mean RR interval and expressed in beats per minute as

(5) $$\mathrm{HR}=\frac{60}{\mathrm{MeanRR}}$$

Together, these statistical RR interval features form a compact and interpretable representation of cardiac rhythm dynamics. Because they rely on temporal variability rather than waveform morphology, these features remain robust across datasets with different acquisition conditions and are well suited for both intra-dataset and cross-dataset ECG rhythm classification. In the current study, however, it is intended to consider only a small number of commonly used time domain RR interval features like mean RR, SDNN, RMSSD, pNN50, and heart rate. The use of these features was motivated by several important factors such as computational efficiency, interpretability, and applicability to short-term recordings of ECG. Despite the practical value of frequency domain and nonlinear HRV descriptors, the robust estimation of these features usually takes more time compared to the features under consideration here.

### 3.5. Feature Ablation Methodology

To investigate the relative contribution of individual RR interval features to rhythm classification performance, a systematic feature ablation analysis is conducted. The Random Forest classifier is first trained using the complete set of statistical RR features, including MeanRR, SDNN, RMSSD, pNN50, and heart rate. Subsequently, a series of experiments is performed in which one feature is removed at a time while keeping all other components of the pipeline unchanged. The model is retrained and evaluated under identical conditions for each ablation setting, enabling direct comparison of performance changes. Variations in classification accuracy and other performance metrics are used to assess the sensitivity of the model to specific RR interval descriptors, providing insights into feature importance and representation robustness.

### 3.6. Random Forest-Based Rhythm Classification

For rhythm-level ECG classification, a Random Forest classifier was employed because of its ability to model nonlinear relationships and interactions among the extracted RR interval features while maintaining relatively low computational complexity. The classifier was trained using the statistical RR interval features derived from the detected R-peaks. Each decision tree in the ensemble was constructed using a bootstrap sample of the training data, and a random subset of features was considered at each split to promote diversity among the trees. The final class prediction was obtained through majority voting across the ensemble.

For the primary binary intra-dataset evaluation on ECG-SDS, the Random Forest classifier was configured with 200 decision trees (n_estimators = 200), a maximum tree depth of 8 (max_depth = 8), and a fixed random seed (random_state = 42). A stratified 70:30 train–test split was used with random_state = 42 to preserve the class distribution between the training and test subsets.

No formal hyperparameter optimization or grid search procedure was performed for the primary intra-dataset Random Forest configuration. Therefore, no separate optimization dataset or performance metric was used to select n_estimators = 200 and max_depth = 8; these values were treated as fixed model settings for the intra-dataset experiments. The held-out ECG-SDS test subset was used to report the performance of this fixed configuration. No explicit class weighting, oversampling, or balanced-sampling procedure was applied in the primary binary intra-dataset experiment. Class distribution was preserved during data partitioning through stratified sampling.

For the classifier-comparison analysis, Random Forest, Support Vector Machine (SVM), K-Nearest Neighbors (KNN), and Logistic Regression were evaluated using the same stratified 70:30 train–test partition (random_state = 42). Random Forest was evaluated directly on the extracted RR-derived features using 200 decision trees (n_estimators = 200), a maximum tree depth of 8 (max_depth = 8), and random_state = 42. For SVM, KNN, and Logistic Regression, the RR-derived feature vectors were standardized using StandardScaler, which was fitted only on the training subset and subsequently applied to the test subset. SVM was configured with an RBF kernel, KNN used five nearest neighbors, and Logistic Regression was configured with a maximum of 1000 iterations and random_state = 42. The corresponding performance results are reported in the revised classifier comparison analysis.

For the cross-dataset binary evaluation, the classifier was fitted using the PhysioNet/CinC 2017 source dataset and subsequently evaluated on ECG-SDS. The implemented Random Forest configuration comprised 500 decision trees, a maximum tree depth of 10, class weights of 1:3 for the normal and abnormal classes, respectively, and random_state = 42. The number of trees was used to provide a sufficiently large ensemble, the maximum depth was used to limit tree complexity, and the class weighting placed greater emphasis on the abnormal class. The random state was fixed at 42 for reproducibility. These hyperparameters were not selected through a formal hyperparameter optimization procedure. ECG-SDS performance did not influence the selection of these Random Forest hyperparameters; its results were considered only in selecting the original decision threshold of 0.35. Feature standardization was fitted using the source data and subsequently applied to the target data. A decision threshold of 0.35 was used as the operating point in the original cross-dataset experiment.

In the original experimental workflow, ECG-SDS prediction results were available when the 0.35 operating threshold was selected; therefore, this threshold was not determined through a source-only validation procedure.

To examine the effect of the decision threshold, a retrospective sensitivity analysis was conducted using only the PhysioNet/CinC 2017 source dataset, without using ECG-SDS for threshold selection in this analysis. A stratified 80:20 training–validation split of the source dataset was used, and the cross-dataset Random Forest configuration was evaluated over thresholds ranging from 0.20 to 0.80 in increments of 0.05. A threshold of 0.55 maximized the abnormal class F1-score on the source-validation subset.

The threshold of 0.35 corresponds to the operating point used in the original cross-dataset analysis. To further assess the influence of threshold selection, an additional retrospective sensitivity analysis was performed using only the PhysioNet/CinC 2017 source dataset.

Although the source-only validation analysis identified 0.55 as the F1-optimal threshold, the threshold of 0.35 yielded higher abnormal class recall in the cross-dataset comparison (74.21% versus 67.19%). Accordingly, the originally reported threshold of 0.35 was retained as the primary operating point, while the results at 0.55 are additionally reported to transparently characterize the trade-off between abnormal rhythm sensitivity, false-positive classifications, and overall classification performance.

Random Forest was selected for the primary analysis because it provided a favourable balance between classification performance, computational efficiency, and interpretability for the selected RR interval feature representation. Its ensemble structure also enables nonlinear interactions among the RR-derived features to be represented without requiring a computationally intensive deep learning architecture.

## 4. Experimental Setup and Evaluation Metrics

### 4.1. Training and Testing Protocol

The proposed ECG rhythm classification framework is evaluated under both intra-dataset and cross-dataset experimental settings. For intra-dataset evaluation, the extracted RR interval features are randomly partitioned into training and testing subsets while maintaining the original class distribution. In contrast, cross-dataset evaluation is performed by training the Random Forest classifier exclusively on the PhysioNet/CinC 2017 ECG dataset and subsequently evaluating it on ECG-SDS, thereby maintaining separation between the records used for model fitting and those used for cross-dataset evaluation. This protocol provides a technical assessment of model transferability under dataset shift. Because ECG-SDS consists of synthetic ECG recordings, the cross-dataset results should not be interpreted as validation in an independent clinical population or as evidence of real-world clinical deployment performance. The retrospective threshold sensitivity analysis used only PhysioNet/CinC 2017 records for model fitting and threshold selection. Following this analysis, the classifier was refitted on the full source dataset and evaluated on ECG-SDS using both decision thresholds. ECG-SDS was not used for fitting the classifier or for selecting the threshold of 0.55 in this retrospective analysis.

### 4.2. Implementation Details

All signal processing, RR feature extraction, and classification experiments were implemented in Python 3.13.15. R-peak detection was performed using NeuroKit2 (version 0.2.13), with nk.ecg_peaks operating under its default method=’neurokit’ setting. The classification models were implemented using Scikit-learn (version 1.6.1).

For the primary intra-dataset ECG-SDS experiment, the Random Forest classifier used 200 decision trees, a maximum tree depth of 8, and random_state = 42. No formal hyperparameter optimization or grid-search procedure was performed for this configuration; therefore, no separate optimization dataset or performance metric was used to select these two hyperparameters. No explicit class weighting, oversampling, or balanced-sampling procedure was applied. Instead, a stratified train–test split was used to preserve the class distribution between the training and test subsets, and class-specific performance metrics were reported to characterize performance under class imbalance.

### 4.3. Evaluation Metrics

Model performance is assessed using multiple complementary metrics to provide a comprehensive evaluation of classification quality. Overall accuracy is reported to measure general correctness, while precision, recall, and F1-score are used to analyze class-specific performance, with particular emphasis on abnormal rhythm detection. To further account for class imbalance, Matthews Correlation Coefficient (MCC) and Cohen’s Kappa coefficient are computed, offering robust measures of agreement beyond chance. In addition, receiver operating characteristic (ROC) analysis is performed, and the area under the ROC curve (AUC) is reported to quantify the discriminative capability of the classifier across varying decision thresholds.

### 4.4. Performance Reporting

Classification results are presented using confusion matrices, ROC curves, and detailed metric tables. A feature ablation analysis is conducted by systematically removing one RR interval feature at a time during training, and the resulting change in classification accuracy is reported to quantify the contribution of individual features. Cross-dataset performance is reported to assess technical transferability under dataset shift, with particular attention to abnormal rhythm recall. Because the target ECG-SDS dataset consists of synthetic recordings, these results are not interpreted as evidence of clinical screening performance or real-world deployment readiness.

## 5. Results

The proposed RR interval–based ECG rhythm classification framework is extensively evaluated under both cross-dataset and same-dataset experimental conditions. To ensure a meaningful and reliable assessment, multiple performance metrics are analyzed, including accuracy, precision, recall, F1-score, area under the ROC curve (AUC), Matthews Correlation Coefficient (MCC), and Cohen’s Kappa coefficient. The evaluation further incorporates feature ablation analysis and physiological validation through RR interval distribution analysis.

### 5.1. Cross-Dataset Performance

**Figure 4:**
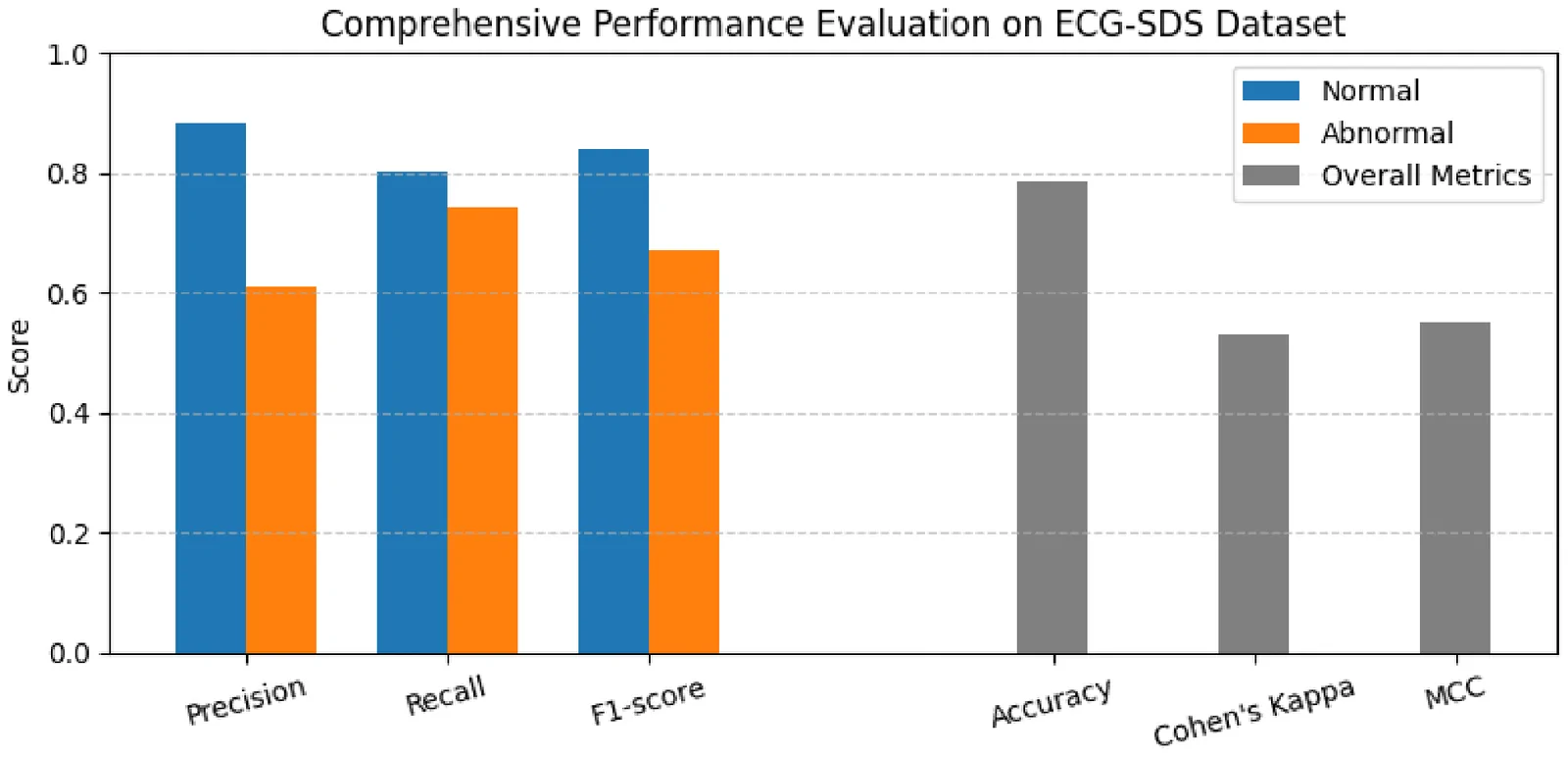
Comprehensive performance evaluation on the cross-dataset, showing class-wise precision, recall, and F1-score for normal and abnormal rhythms, along with overall accuracy, Cohen’s Kappa, and Matthews Correlation Coefficient (MCC).

The overall cross-dataset classification performance is illustrated in Figure 4, with detailed numerical results reported in Table 1. When trained on the PhysioNet/CinC 2017 dataset and evaluated on the synthetic ECG-SDS target dataset, the proposed model achieved an overall accuracy of 78.6%. To quantify the statistical uncertainty associated with this cross-dataset performance, a bootstrap analysis with 1000 resamples of the ECG-SDS target dataset was additionally performed. The resulting 95% bootstrap confidence interval for accuracy was 76.47–80.73%. Because ECG-SDS consists of synthetic ECG recordings, these results represent a technical assessment of transferability between the evaluated datasets and should not be interpreted as validation in an independent clinical population or as evidence of clinical deployment performance.

The influence of the decision threshold was further examined by comparing the originally reported threshold of 0.35 with an alternative threshold of 0.55 identified through retrospective source-only validation. Using the same Random Forest configuration refitted on the complete PhysioNet/CinC 2017 dataset, the threshold of 0.35 reproduced the reported cross-dataset accuracy of 78.60%, with an abnormal class precision of 61.31%, recall of 74.21%, and F1-score of 0.6714. In comparison, the alternative threshold of 0.55 achieved an accuracy of 88.67%, abnormal class precision of 92.24%, recall of 67.19%, and F1-score of 0.7775. The threshold of 0.35 correctly identified 328 of the 442 abnormal recordings, compared with 297 at the threshold of 0.55, thereby detecting 31 additional abnormal recordings. However, the number of false-positive classifications increased from 25 at the threshold of 0.55 to 207 at the threshold of 0.35. These findings demonstrate that the threshold of 0.35 yielded a higher-sensitivity operating point in this comparison, whereas 0.55 improved precision, F1-score, and overall accuracy at the expense of abnormal class recall.

To assess whether differences in recording duration influenced the cross-dataset results, an additional sensitivity analysis was performed using a harmonized 10 s source-data window. PhysioNet/CinC 2017 recordings shorter than 10 s were excluded, and the first 10 s were retained from each remaining recording. Following RR feature eligibility screening, 8485 source recordings were retained for this analysis. The classifier was retrained using these harmonized 10 s recordings and evaluated on the same ECG-SDS test set using the same cross-dataset decision protocol. The 10 s analysis yielded an accuracy of 68.13% and an ROC-AUC of 0.8476, compared with 78.60% accuracy and an ROC-AUC of 0.8363 in the full-duration analysis. Abnormal class recall increased from 74.2% to 81.9%. The corresponding confusion matrix was [[660, 398], [80, 362]], indicating that the reduction in overall accuracy was primarily associated with an increase in false-positive abnormal predictions among normal recordings. These findings show that harmonizing source-recording duration materially affected threshold-dependent classification performance, whereas discriminative performance measured by ROC-AUC remained comparable.

To assess the potential influence of treating technically noisy recordings as rhythm abnormalities, an additional sensitivity analysis was performed after excluding all 283 retained noisy (∼) PhysioNet/CinC 2017 recordings from model development. The classifier was retrained using the remaining 8240 source recordings, comprising 5049 Normal, 738 Atrial Fibrillation, and 2453 Other recordings, and was evaluated on the same ECG-SDS target dataset using the same cross-dataset decision protocol. Exclusion of the noisy recordings resulted in an accuracy of 79.47%, compared with 78.60% when the noisy recordings were included. The ROC-AUC remained essentially unchanged (0.8361 versus 0.8363), and abnormal class recall was also essentially unchanged (74.21% versus 74.2%). The abnormal class F1-score increased from 0.671 to 0.681. These results indicate that, in this sensitivity analysis, exclusion of the noisy source recordings produced only modest changes in the overall cross-dataset performance.

The binary abnormal class performance was further examined separately for the two ECG-SDS abnormal rhythm subgroups. All 219 atrial fibrillation recordings were classified as abnormal, corresponding to a sensitivity of 100.0%. In contrast, 109 of the 223 sinus tachy–brady recordings were classified as abnormal, corresponding to a sensitivity of 48.88%. Together, 328 of the 442 abnormal ECG-SDS recordings were correctly identified, yielding the overall abnormal class sensitivity of 74.21%. Thus, the combined abnormal class sensitivity masks substantial variation in detection performance between the two component rhythm subgroups.

Although the cross-dataset evaluation accuracy is lower than the corresponding same-dataset performance, the observed reduction is consistent with differences in acquisition characteristics, rhythm composition, annotation schemes, and signal properties between the two datasets. Because the target ECG-SDS dataset consists of synthetic ECG recordings, these results should be interpreted specifically as a technical assessment of cross-dataset transferability between the evaluated datasets and should not be considered evidence of generalization to an independent clinical population. For the normal rhythm class, precision and recall values of 0.882 and 0.804, respectively, yield an F1-score of 0.841, indicating reliable identification of normal cardiac rhythms.

For abnormal rhythms, the model achieves a precision of 0.613 and a recall of 0.742, corresponding to an F1-score of 0.671. The abnormal class recall of 0.742 indicates that the model retained sensitivity to the abnormal rhythm categories represented in the synthetic ECG-SDS dataset under cross-dataset evaluation. The macro-averaged F1-score of 0.756 and weighted F1-score of 0.791 further confirm balanced performance despite class imbalance. In addition, the MCC value of 0.520 and Cohen’s Kappa coefficient of 0.515 indicate moderate agreement between the predicted and true labels beyond chance.

The observed performance reduction between same-dataset and cross-dataset evaluation further highlights the significant impact of ECG domain shift on machine learning-based rhythm classification systems. The bootstrap confidence interval indicates that the observed cross-dataset accuracy is associated with measurable variability across bootstrap resamples of the ECG-SDS target dataset.

**Figure 5:**
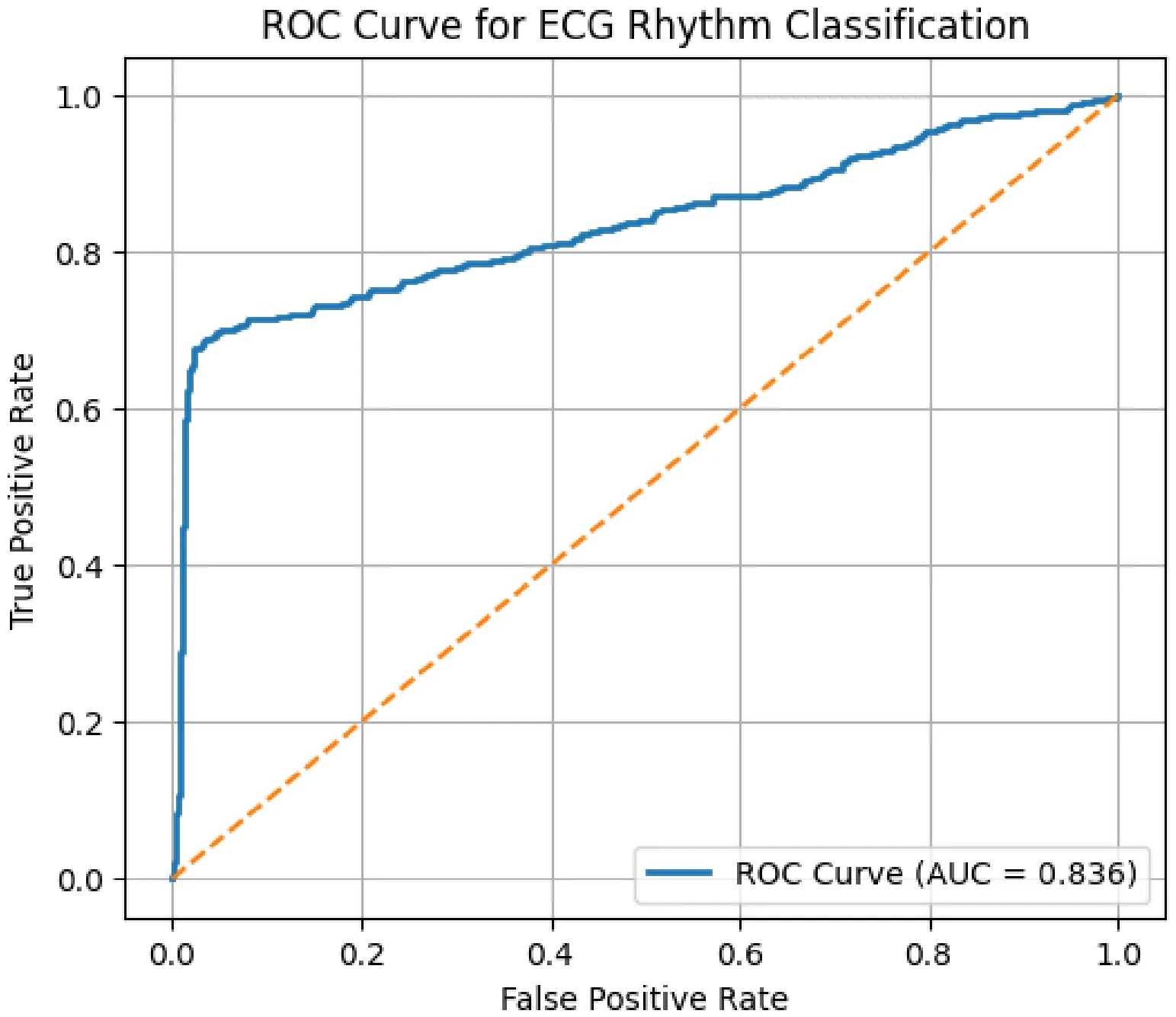
Receiver operating characteristic (ROC) curve for cross-dataset ECG rhythm classification. The solid blue line represents the ROC curve of the proposed classification model (AUC = 0.836), while the orange dashed diagonal line indicates chance-level classification performance (AUC = 0.5).

**Figure 6:**
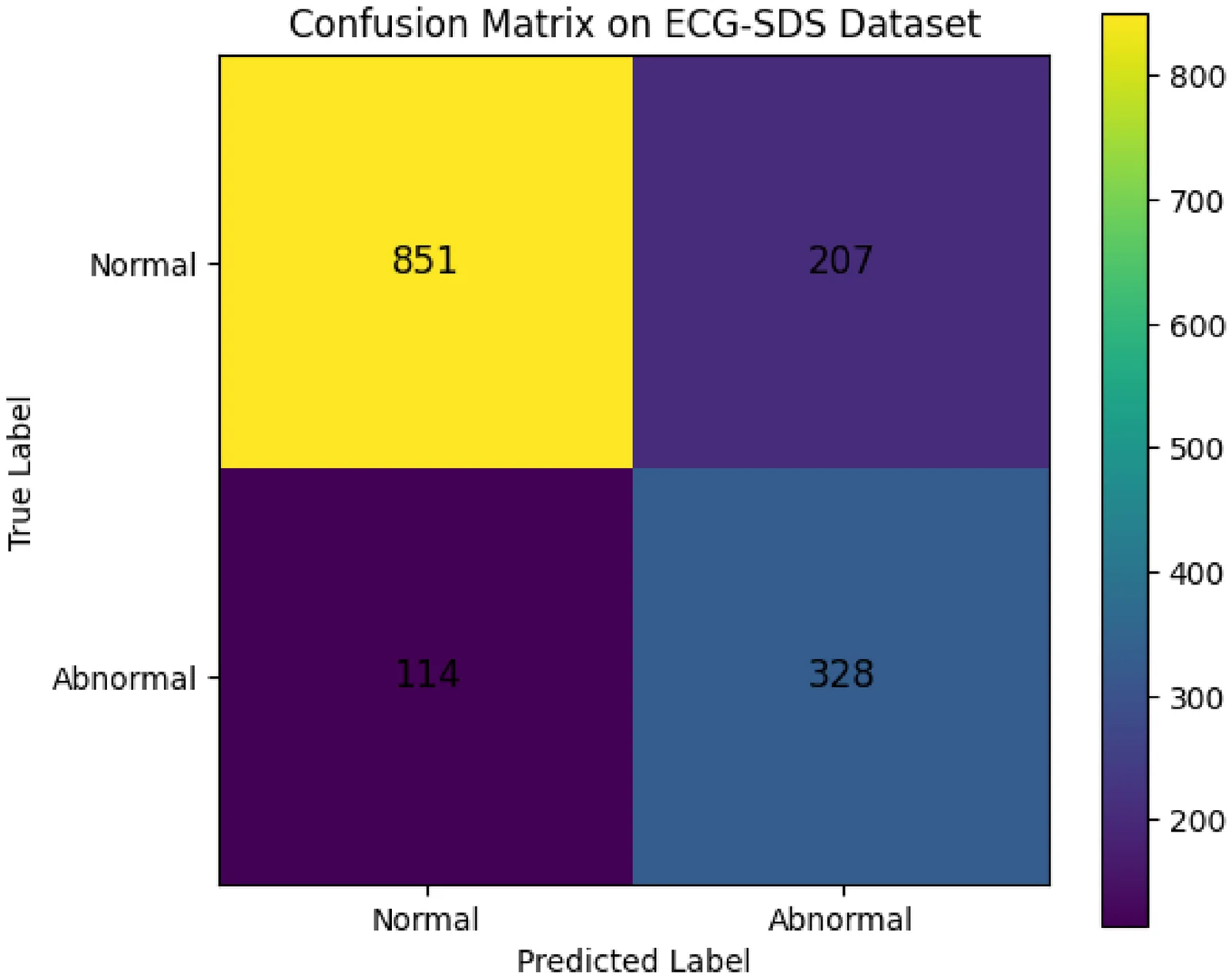
Confusion matrix for cross-dataset ECG rhythm classification on the ECG-SDS dataset.

The discriminative capability of the classifier under cross-dataset conditions is further validated by the ROC curve shown in Figure 5, which yields an AUC of approximately 0.836. More precisely, the observed AUC was 0.8363, with a 95% bootstrap confidence interval of 0.8072–0.8632. This interval provides an estimate of the uncertainty associated with the discriminative performance on the ECG-SDS target dataset. The corresponding confusion matrix in Figure 6 shows that most normal rhythm samples are correctly classified, while the majority of abnormal rhythm samples are also detected, with misclassifications primarily occurring between rhythm categories exhibiting overlapping temporal characteristics.

Overall, these findings indicate that the RR interval statistical representation retains meaningful discriminative information under cross-dataset evaluation. However, because ECG-SDS prediction results were considered when selecting the original decision threshold of 0.35, the corresponding threshold-dependent results should not be interpreted as performance on an entirely untouched target dataset. The reduction in performance relative to same-dataset evaluation may reflect dataset shift as well as differences in model configuration and evaluation protocol. The reported bootstrap confidence intervals provide a more transparent characterization of the uncertainty associated with the cross-dataset performance. Because ECG-SDS consists of synthetic ECG recordings, these findings should be interpreted as a technical assessment of transferability between the evaluated datasets rather than as evidence of generalization to an independent clinical population.

### 5.2. Intra-Dataset Performance

**Figure 7:**
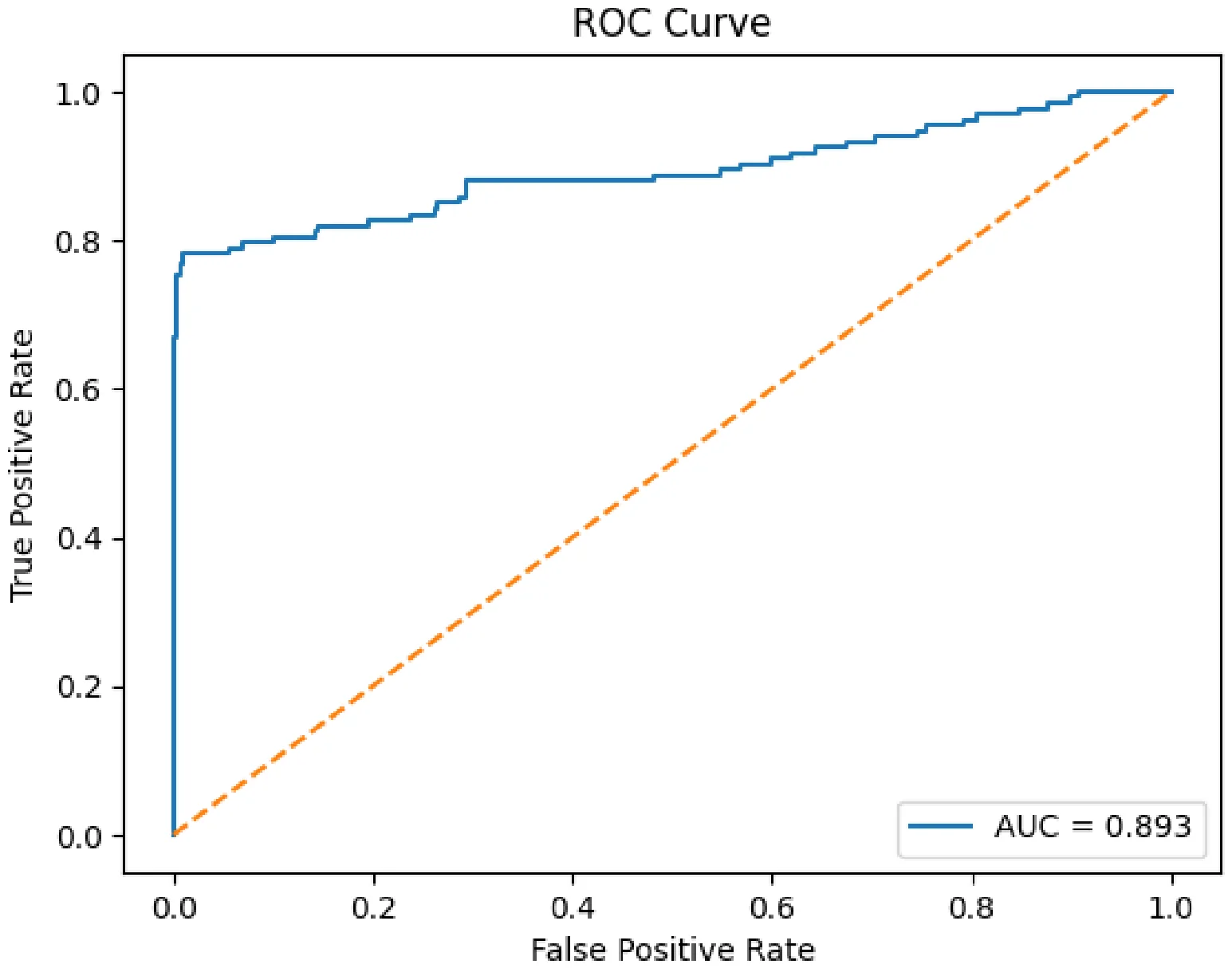
ROC for intra-dataset evaluation on the ECG-SDS dataset.

To establish an upper-bound performance benchmark, the proposed framework is evaluated under same-dataset conditions using the ECG-SDS dataset for both training and testing. The ROC curve shown in Figure 7 demonstrates a substantially improved AUC of 0.893, indicating excellent class separability in the absence of domain shift. The detailed classification results summarized in Table 2 reveal an overall accuracy of 93%. To assess the stability of the same-dataset performance across different data partitions, an additional 10-fold stratified cross-validation analysis was performed on the ECG-SDS dataset. Across the 10 folds, the proposed framework achieved a mean accuracy of 92.20% ± 2.63% and a mean AUC of 0.8880 ± 0.0477. These results provide an estimate of performance variability across different stratified data partitions.

For the normal rhythm class, the model achieves a precision of 0.92 and an exceptionally high recall of 0.99, resulting in an F1-score of 0.95. For abnormal rhythms, precision and recall values of 0.97 and 0.78, respectively, yield an F1-score of 0.87. These results indicate highly reliable abnormal rhythm detection while maintaining a low false-positive rate.

### 5.3. Exploratory Multi-Class Evaluation

To further investigate the scalability of the proposed RR-based statistical descriptors beyond binary abnormal rhythm screening, an exploratory multi-class evaluation was conducted under the same-dataset setting using ECG-SDS. The three ECG-SDS rhythm categories—normal sinus rhythm, atrial fibrillation (AFib), and sinus tachy–brady rhythm—were retained according to their original dataset annotations. The proposed framework achieved an overall classification accuracy of 91.67%, as shown in Table 3, demonstrating its ability to discriminate among the three rhythm categories under a controlled same-dataset setting.

For this exploratory multi-class experiment, the ECG-SDS dataset comprised 1500 recordings and was partitioned into training and test subsets using a stratified 80:20 split with random_state = 42, resulting in 1200 training recordings and 300 test recordings. Stratification was performed according to the three-class rhythm labels to preserve the class distribution across the training and test subsets. Five RR interval-derived features, namely mean RR, SDNN, RMSSD, pNN50, and heart rate, were used as classifier inputs. The Random Forest classifier comprised 100 decision trees with random_state = 42, while the remaining parameters were retained at their default scikit-learn settings. The reported accuracy of 91.67% was obtained from this single stratified train–test split and therefore does not represent an average across repeated evaluations.

**Figure 8:**
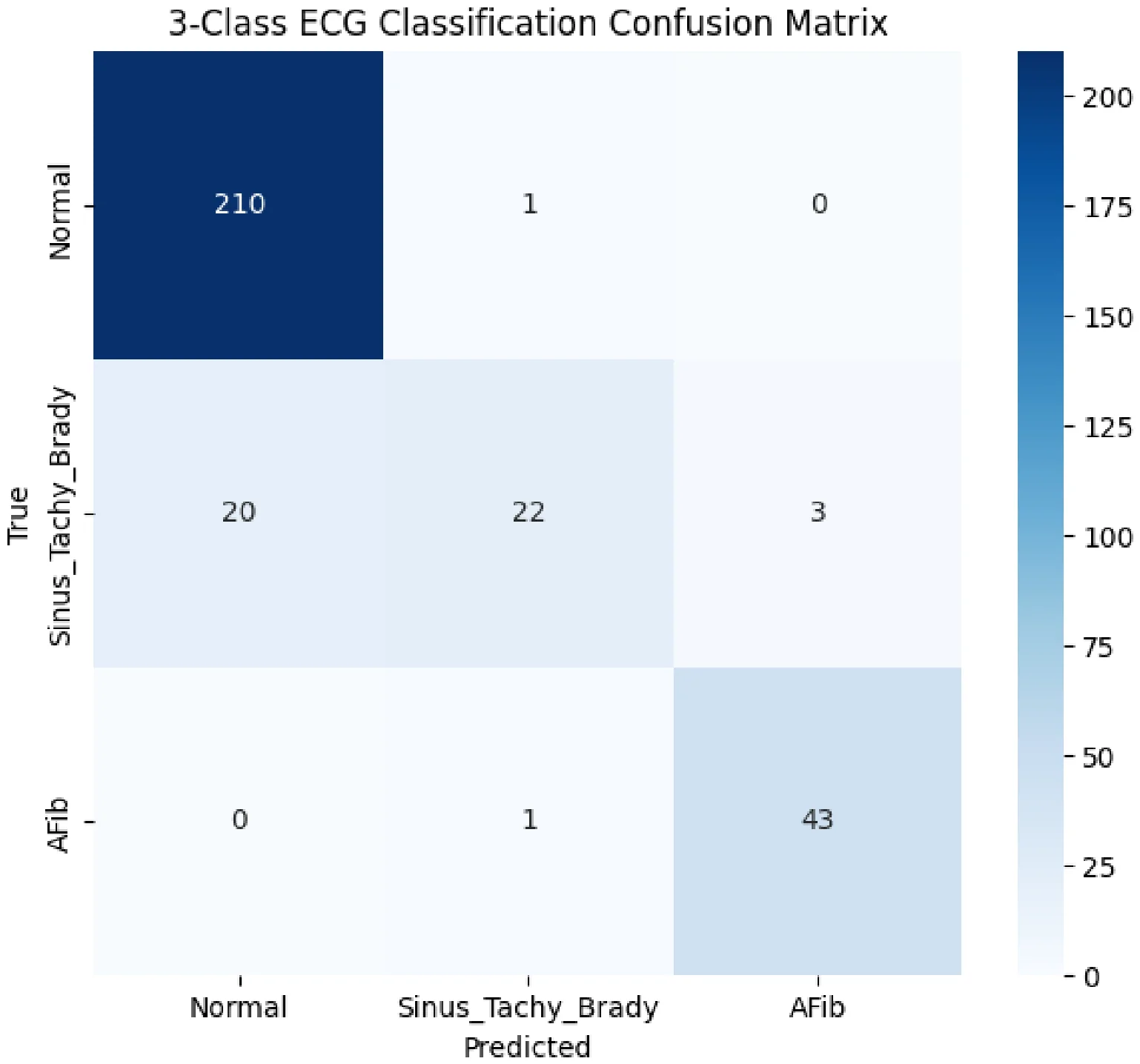
Confusion matrix for the exploratory three-class same-dataset ECG rhythm classification experiment using a single stratified 80:20 train–test split. The test set comprised 211 Normal, 45 Sinus Tachy–Brady, and 44 AFib recordings.

In the exploratory three-class same-dataset analysis, the Random Forest achieved an overall accuracy of 91.67% (275/300) on the single stratified test split. The test set comprised 211 Normal, 45 Sinus Tachy–Brady, and 44 AFib recordings. The Normal class achieved a precision of 0.91, recall of 1.00, and F1-score of 0.95, while the AFib class achieved a precision of 0.93, recall of 0.98, and F1-score of 0.96. Performance was lower for the Sinus Tachy–Brady class, with a precision of 0.92, recall of 0.49, and F1-score of 0.64. The macro-averaged F1-score was 0.85, whereas the weighted-averaged F1-score was 0.91. The corresponding confusion matrix is shown in Figure 8.

As shown in Figure 8, 210 of the 211 Normal recordings and 43 of the 44 AFib recordings were correctly classified. In contrast, 22 of the 45 Sinus Tachy–Brady recordings were correctly identified, while 20 were classified as Normal and 3 as AFib. The confusion matrix therefore indicates that the reduced class-wise performance was primarily associated with the Sinus Tachy–Brady class.

Cross-dataset multi-class evaluation was not considered because the PhysioNet/CinC 2017 and ECG-SDS datasets use different rhythm taxonomies that do not permit a clinically meaningful one-to-one mapping of all classes. In particular, the heterogeneous “Other” category in the PhysioNet/CinC 2017 dataset is not clinically equivalent to the specifically defined sinus tachy–brady category in ECG-SDS. Therefore, multi-class evaluation was restricted to the same-dataset ECG-SDS setting, where the class definitions are consistent between the training and test subsets. Cross-dataset generalization was evaluated using the binary normal-versus-abnormal formulation, for which the rhythm categories could be consistently mapped across the two datasets.

### 5.4. Feature-Level Sensitivity Analysis

The feature-level sensitivity analysis was performed using the same 70:30 stratified train–test partition employed for the primary same-dataset binary evaluation. To ensure that the perturbation magnitude was comparable across features with different numerical scales, the five RR-derived features were standardized using statistics estimated from the training set, and the same transformation was applied to the test set. A Random Forest classifier was trained on the standardized training features, and zero-mean Gaussian perturbations were subsequently added to the standardized test feature vectors without retraining the classifier. Perturbation standard deviations of 0.00, 0.02, 0.05, 0.10, and 0.20 were evaluated, as shown in Table 4. At zero perturbation, the accuracy was 92.89%, consistent with the 93% accuracy reported for the primary same-dataset binary evaluation. The corresponding accuracies at perturbation levels of 0.02, 0.05, 0.10, and 0.20 were 93.11%, 92.67%, 91.78%, and 90.67%, respectively. These results indicate that the classifier remained relatively stable under small feature-level perturbations, with a modest reduction in accuracy as the perturbation magnitude increased. The reported results correspond to a single reproducible perturbation realization and should therefore be interpreted as a feature-level sensitivity analysis rather than as an evaluation of robustness to noise in the raw ECG signal or the complete ECG-processing pipeline.

### 5.5. Feature Ablation Analysis

**Figure 9:**
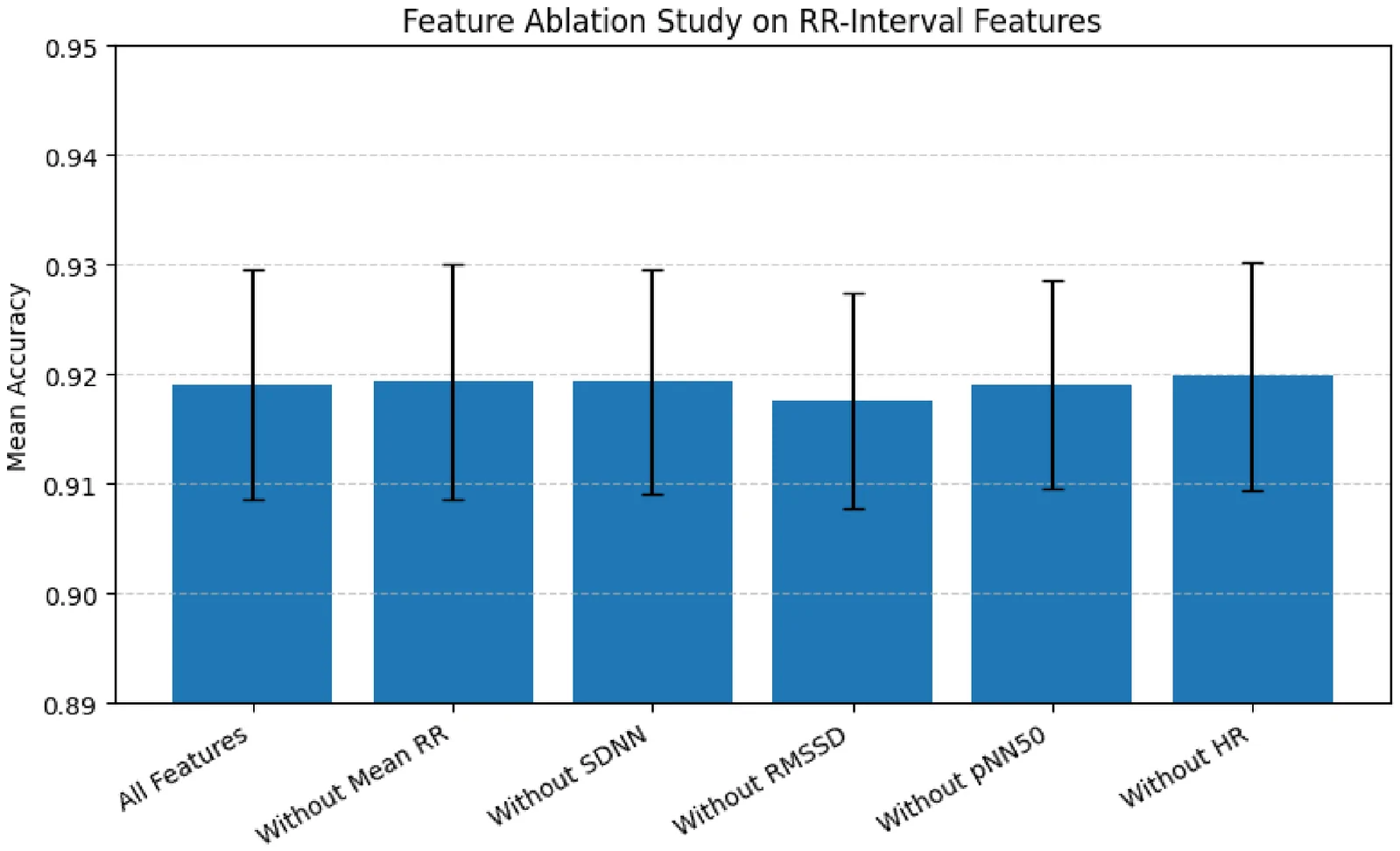
Feature ablation analysis using 10 independent stratified train–test splits. Bars represent mean accuracy, and error bars indicate the standard deviation across repeated splits.

The contribution and stability of individual RR interval features were further examined through a systematic feature ablation study using 10 independent stratified train–test splits. The resulting mean accuracies and standard deviations are reported in Table 5, while the corresponding accuracy variations are illustrated in Figure 9. With all five RR interval features retained, the model achieved a mean accuracy of 91.91% ± 1.05%. Removing individual features resulted in only small changes in mean accuracy, with values of 91.93% ± 1.08% without mean RR, 91.93% ± 1.02% without SDNN, 91.76% ± 0.98% without RMSSD, 91.91% ± 0.95% without pNN50, and 91.98% ± 1.04% without HR. These results indicate that the performance differences among the feature configurations are small relative to the variability observed across repeated data splits.

Statistical comparisons between the all-feature configuration and each individual feature-removed configuration were performed using a paired two-sided *t*-test (scipy.stats.ttest_rel). The accuracies obtained from the same 10 stratified train–test splits were paired between the baseline and each corresponding ablated configuration. The resulting *p*-values were 0.5911, 0.8473, 0.0445, 1.0000, and 0.1934 for the removal of mean RR, SDNN, RMSSD, pNN50, and HR, respectively. Because five individual feature removal comparisons were performed, the nominal *p*-value of 0.0445 observed for RMSSD should be interpreted cautiously in the context of multiple comparisons and should not be regarded as strong evidence of an independent feature effect. Moreover, the associated change in mean classification accuracy was small. Overall, the ablation results do not provide strong evidence that removal of any single feature produces a practically meaningful change in classification performance. Because the repeated evaluations were generated from different stratified splits of the same underlying dataset, the resulting performance estimates are not necessarily statistically independent, as individual recordings may occur in overlapping training or test subsets across splits. Consequently, the paired-test results and associated nominal *p*-values should be interpreted as exploratory rather than as confirmatory statistical evidence.

Mean RR and heart rate are deterministically related in the implemented feature formulation, since heart rate was calculated as 60/MeanRR. Therefore, these two variables should be regarded as redundant rather than fully complementary descriptors. To assess their joint contribution, an additional grouped ablation was performed in which mean RR and HR were removed simultaneously, leaving SDNN, RMSSD, and pNN50 as the input features. Using the same 10 repeated stratified train–test splits, the grouped-ablation configuration achieved a mean accuracy of 84.64%±1.39%, compared with 91.91%±1.05% for the complete five-feature configuration, corresponding to a reduction of approximately 7.27 percentage points. In contrast to the negligible changes observed when either mean RR or HR was removed individually, their simultaneous removal therefore produced a substantial decrease in classification accuracy. This result indicates that information related to the average RR interval contributes meaningfully to classification, while mean RR and HR themselves encode redundant information.

The feature ablation experiments also showed that removing individual RR-derived descriptors resulted in only small changes in classification accuracy. Although the removal of RMSSD yielded a nominal paired *p*-value of 0.0445, this result should be interpreted cautiously because five individual feature removal comparisons were performed, and the associated difference in classification accuracy was small. These results suggest that the classification performance depends on the combined information provided by the feature set rather than on a single dominant descriptor. Therefore, the individual feature importance results should not be interpreted as evidence that any one feature is solely responsible for the observed classification performance.

### 5.6. Illustrative RR Interval Distribution Analysis

**Figure 10:**
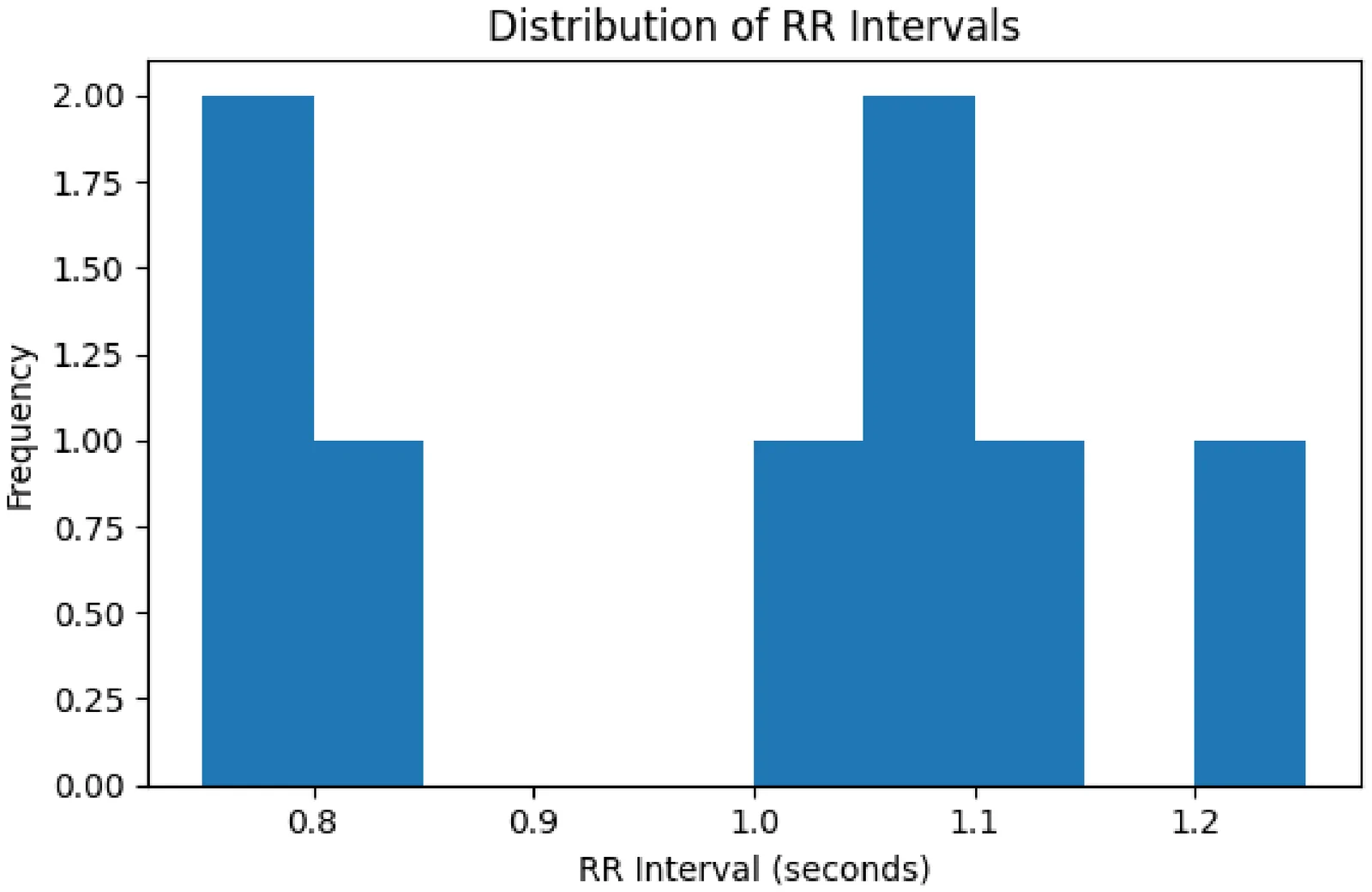
Histogram showing the distribution of RR intervals in the illustrative ECG sample. The horizontal axis represents RR interval duration in seconds, while the vertical axis represents the corresponding frequency of occurrence.

The physiological relevance of RR intervals was examined through their distribution, as illustrated in Figure 10. RR intervals represent the time between consecutive R-peaks and provide a direct measure of beat-to-beat timing. Consequently, variation in RR interval duration provides a physiologically interpretable representation of temporal heart rhythm dynamics.

Figure 10 presents a histogram of RR interval values, with RR interval duration in seconds on the horizontal axis and frequency of occurrence on the vertical axis. The distribution illustrates the occurrence of different beat-to-beat intervals within the evaluated ECG sample and provides a direct visualization of temporal variation in successive heartbeats. Such variation forms the basis for the statistical RR interval descriptors used in the proposed classification framework, including mean RR, SDNN, RMSSD, pNN50, and heart rate.

The histogram is intended as an illustrative visualization of RR interval distribution rather than as a class-specific comparison. Therefore, no conclusions regarding class-specific medians, interquartile ranges, or distributional separation are drawn from Figure 10. The classification experiments and feature analyses reported elsewhere in the manuscript provide the quantitative assessment of the discriminative information contained in the RR-derived feature representation.

The classification results further illustrate how the proposed framework performs under different evaluation settings. In the same-dataset evaluation on ECG-SDS, the framework achieved accuracies of 93.0% and 91.67% for binary and multi-class classification, respectively. In the cross-dataset setting, where the model was trained on PhysioNet/CinC 2017 and evaluated on the synthetic ECG-SDS dataset, the framework achieved an accuracy of 78.6% for binary classification. The lower performance observed under cross-dataset evaluation may reflect dataset shift as well as differences in model configuration and evaluation protocol. Therefore, the observed performance difference should not be attributed exclusively to dataset shift.

The comparison with existing studies presented in Table 6 is provided for context rather than as a direct performance ranking. The studies considered use different datasets, class definitions, feature representations, classifiers, and evaluation protocols. Notably, the reported results from most existing studies are based on intra-dataset evaluation, while the proposed framework also includes independent cross-dataset evaluation. Therefore, the reported accuracy values should not be considered directly comparable across studies. Instead, Table 6 provides a broader view of the performance reported in ECG arrhythmia classification and emphasizes the difference between controlled same-dataset evaluation and external cross-dataset generalization.

Overall, the consistency between the physiological patterns observed in the RR interval distributions and the classification results supports the use of RR interval features as an interpretable and computationally efficient representation for ECG rhythm classification. The cross-dataset experiments also show that the proposed representation retains useful rhythm-related information under dataset shift, while highlighting the challenges associated with generalization across heterogeneous ECG data sources.

### 5.7. Feature Importance Analysis

The feature importance scores provided by the Random Forest classifier further elucidate the role of individual RR interval-based characteristics. According to Table 7, the most important characteristic turned out to be RMSSD, followed by SDNN and heart rate. Such a choice seems physiologically justified as both RMSSD and SDNN reflect variations in heartbeat rhythm over short and long periods of time, and they are closely related to arrhythmia such as atrial fibrillation. The mean RR interval and pNN50 were also important for the classification but had relatively low feature importance scores compared to other characteristics. Importantly, the distribution of the feature importance scores reveals that the proposed approach was not based on only one feature but involved several features based on RR intervals. The relationship between mean RR and heart rate was also taken into account when interpreting the feature-importance results. Since heart rate is inversely related to the mean RR interval, the two descriptors contain partially overlapping information and may affect the distribution of Random Forest feature importance when used together. Therefore, the feature-importance values should be viewed as indicators of the usefulness of the overall feature representation rather than as evidence of independent physiological contributions. Furthermore, the feature-importance analysis used the impurity-based feature-importance measure of Random Forest and individual importance rankings should consequently be interpreted with caution.

### 5.8. Comparison of Classifiers

To ensure a consistent comparison among classifiers, Random Forest, Support Vector Machine (SVM), K-Nearest Neighbors (KNN), and Logistic Regression were re-evaluated using the same stratified 70:30 train–test partition (random_state = 42) and the same feature preprocessing protocol. As shown in Table 8, Random Forest achieved the highest overall accuracy of 92.89% and the highest F1-score of 0.8667 among the evaluated classifiers. SVM and Logistic Regression achieved higher precision but lower recall, whereas KNN provided comparatively balanced performance but remained below Random Forest in both accuracy and F1-score. These results support the selection of Random Forest for the primary binary classification experiment under the common evaluation protocol.

## 6. Discussion

The experimental results indicate that statistical RR interval features can provide a compact and interpretable representation for ECG rhythm abnormality classification. In the same-dataset evaluation on ECG-SDS, the proposed framework achieved an overall accuracy of 93.0%. This finding suggests that a relatively small set of RR interval descriptors can capture useful temporal information associated with cardiac rhythm without relying on a computationally intensive deep learning architecture. However, this performance was obtained under a controlled same-dataset setting and should not be taken as representative of performance on an independent clinical dataset.

Following a consistent evaluation protocol, the classifier-comparison experiment was conducted using the same stratified 70:30 train–test partition (random_state = 42) and feature preprocessing procedure as the primary binary intra-dataset evaluation. Under this setting, Random Forest achieved an accuracy of 92.89%, which corresponds to approximately 93.0% when rounded. Thus, the Random Forest result in the classifier-comparison analysis is consistent with the accuracy reported for the primary binary intra-dataset evaluation.

To assess cross-dataset transferability, a cross-dataset experiment was conducted by training the model on the PhysioNet/CinC 2017 dataset and testing it on the ECG-SDS dataset, which consists of synthetic ECG recordings. The framework achieved an accuracy of 78.6%, with an abnormal rhythm recall of 74.2% and a weighted F1-score of 0.79. Bootstrap analysis resulted in a 95% confidence interval of 76.47–80.73% for accuracy, while the AUC was 0.8363 with a 95% confidence interval of 0.8072–0.8632. The lower performance observed in the cross-dataset experiment may reflect dataset shift as well as differences in model configuration and evaluation protocol. Differences in recording duration, signal characteristics, rhythm distributions, annotation schemes, class weighting, and decision threshold may also have contributed to the observed performance difference.

The additional sensitivity analysis excluding the 283 retained noisy PhysioNet/CinC 2017 recordings showed that their inclusion had only a modest effect on the overall cross-dataset results. After exclusion of these recordings, accuracy increased from 78.60% to 79.47%, while ROC-AUC remained essentially unchanged (0.8363 versus 0.8361), and abnormal class recall remained approximately 74.2%. This finding indicates that the principal cross-dataset performance was not strongly dependent on inclusion of the technically noisy source recordings in this sensitivity analysis. However, subgroup analysis of the ECG-SDS abnormal class revealed substantial heterogeneity: sensitivity was 100.0% for atrial fibrillation (219/219) but 48.88% for sinus tachy–brady rhythm (109/223). Therefore, the combined abnormal class recall of 74.21% should not be interpreted as indicating uniform performance across the component abnormal rhythm subgroups.

It is important to note that the synthetic nature of ECG-SDS limits the clinical interpretation of this cross-dataset experiment. Therefore, these results should be interpreted as a technical assessment of transferability across datasets rather than as validation in an independent clinical population. Evaluation using independent real-world clinical ECG cohorts is required before conclusions regarding clinical generalizability can be drawn.

The main focus of this work is not to develop a new classification algorithm but to examine whether a small set of physiologically meaningful RR interval descriptors can retain useful rhythm information across different datasets. The cross-dataset results suggest that the temporal information captured by these features retains some discriminative capability when the model trained on PhysioNet/CinC 2017 is applied to the synthetic ECG-SDS dataset. At the same time, the lower performance observed in the cross-dataset setting is consistent with the challenges associated with transfer across heterogeneous datasets. However, because the same-dataset and cross-dataset experiments used different model configurations and evaluation protocols, the observed performance difference cannot be attributed exclusively to dataset shift. Differences in model configuration, class weighting, decision threshold, and evaluation protocol may also have contributed to the observed performance gap. These findings therefore demonstrate both the potential and the limitations of RR interval statistics for technical cross-dataset transferability. Because ECG-SDS consists of synthetic ECG recordings, these results should not be interpreted as evidence of generalization to an independent clinical population.

RR interval features also provide a relatively transparent representation, as their values are directly related to beat-to-beat cardiac timing. Unlike morphology-based features, they primarily describe temporal variation between successive heartbeats and may therefore be less sensitive to certain waveform- or amplitude-related changes. However, this does not imply that the representation is unaffected by signal quality. Reliable R-peak detection is essential because errors in identifying the peaks directly affect the calculated RR intervals, which can subsequently influence all derived statistical features and the final classification.

The exploratory multi-class experiment provides further insight into the capability and limitations of the proposed RR-based representation. This analysis was restricted to the same-dataset ECG-SDS setting, in which the original dataset-specific classes of Normal, AFib, and Sinus Tachy–Brady were retained. The results indicate that RR interval descriptors can capture useful rhythm-related temporal information under a common dataset setting. However, the multi-class findings should be interpreted as exploratory, particularly because ECG-SDS consists of synthetic ECG recordings. A cross-dataset multi-class evaluation was not retained because the rhythm taxonomies of PhysioNet/CinC 2017 and ECG-SDS do not provide a clinically justified one-to-one mapping, particularly between the heterogeneous PhysioNet/CinC 2017 Other category and the ECG-SDS Sinus Tachy–Brady category.

The feature ablation results showed that removing individual RR descriptors resulted in only small changes in classification performance. Across the repeated stratified train–test splits, the differences between the complete feature set and the individual feature removal settings were generally small relative to the variation observed between splits. RMSSD and SDNN showed relatively higher feature importance, but the ablation results do not indicate that either feature alone is responsible for the overall performance. Instead, the findings suggest that the selected feature set collectively captures useful information about beat-to-beat timing. Mean RR and heart rate are deterministically related in the implemented formulation because heart rate is calculated as 60/MeanRR; therefore, these two variables should be regarded as redundant rather than complementary. Consistent with this relationship, their simultaneous removal reduced the mean classification accuracy from 91.91% to 84.64%.

Among the evaluated classifiers, Random Forest provided a favourable balance between classification performance, computational cost, and interpretability for the selected feature representation. Its ability to capture nonlinear relationships and interactions among a small number of statistical features makes it suitable for the present setting. However, this finding is specific to the datasets and experimental conditions considered in this study and should not be interpreted as evidence that Random Forest is universally the best classifier for RR-based ECG analysis.

The feature-level sensitivity analysis showed that classification performance remained relatively stable under small Gaussian perturbations and gradually decreased as the perturbation magnitude increased. The baseline accuracy at a perturbation level of 0.00 was 92.89%, while accuracies of 93.11%, 92.67%, 91.78%, and 90.67% were obtained at perturbation levels of 0.02, 0.05, 0.10, and 0.20, respectively. In this experiment, zero-mean Gaussian perturbations were applied to the standardized test feature vectors after RR interval feature extraction, rather than to the raw ECG signals. Therefore, this experiment should be interpreted specifically as a feature-level sensitivity analysis. It assesses the sensitivity of the classifier to perturbations in the extracted feature representation and does not evaluate the robustness of the R-peak detection procedure, the complete ECG-processing pipeline, or the effects of physiological and acquisition-related noise in raw ECG recordings.

Several limitations should be considered. The framework relies on accurate R-peak detection, and errors at this stage may affect all subsequent RR-based features. In addition, the current feature set is primarily limited to time-domain RR statistics and does not capture ECG morphology or the wider range of frequency-domain and nonlinear HRV information. The two datasets follow different rhythm taxonomies; therefore, multi-class evaluation was restricted to the same-dataset ECG-SDS setting rather than performing an unsupported cross-dataset multi-class comparison.

Recording duration represents an additional limitation of the present analysis. Although RR-derived variability measures can be calculated from short RR sequences, estimates obtained from very short ECG recordings may have limited stability and physiological interpretability and should not be considered equivalent to conventional HRV assessment based on longer recordings. In the present sensitivity analysis, restricting the PhysioNet/CinC 2017 source recordings to a harmonized 10 s window reduced cross-dataset accuracy from 78.60% to 68.13%, while the ROC-AUC remained comparable (0.8363 for the full-duration analysis versus 0.8476 for the 10 s analysis). These findings indicate that differences in recording duration and in the resulting number of available RR intervals can influence cross-dataset performance and should therefore be considered when interpreting the reported transferability results.

An important limitation is that ECG-SDS consists of synthetic ECG recordings rather than recordings acquired from an independent clinical population. Consequently, the cross-dataset experiment provides a technical assessment of transferability and cannot establish clinical generalizability across independent patient cohorts or real-world acquisition environments. Although repeated stratified splits and bootstrap confidence intervals were used in selected experiments, evaluation using independent real-world clinical ECG datasets is required to provide stronger evidence of generalizability.

From a practical perspective, the low computational cost and relatively clear physiological interpretation of RR interval features make them potentially useful for lightweight ECG decision support systems. However, such applications would require further validation using larger and more heterogeneous clinical datasets. Future evaluations should consider different patient populations, recording devices, acquisition conditions, and clinically verified annotations. Assessment of calibration, false-negative behaviour, reliability, and the potential consequences of automated decisions would also be important before considering clinical use.

Future work will investigate the incorporation of frequency-domain and nonlinear HRV features, improved feature selection methods, domain adaptation techniques, and lightweight hybrid models. Evaluating the framework across multiple independent and multi-centre ECG databases will also help assess how well the approach generalizes under varying acquisition conditions and across different patient populations.

Overall, the results suggest that statistical RR interval descriptors can provide a compact and relatively interpretable basis for ECG rhythm classification. The framework showed good performance under same-dataset evaluation and retained useful discriminative ability during cross-dataset testing on the synthetic ECG-SDS dataset, although with a noticeable reduction in performance. These findings support the technical feasibility of RR-based ECG classification and cross-dataset transferability, while broader evaluation using independent real-world clinical datasets is required before claims regarding clinical generalizability, utility, or deployment can be made.

Although the proposed approach shows technical feasibility for RR interval-based rhythm classification, the current results should not be considered evidence of clinical validation or immediate readiness for clinical screening or deployment. In particular, ECG-SDS consists of synthetic ECG recordings, and the cross-dataset evaluation therefore represents a technical assessment of transferability rather than validation in an independent clinical population. Establishing clinical applicability would require validation using larger and more diverse real-world clinical datasets, prospective evaluation, assessment under different acquisition conditions, and comparison with established clinical workflows. Therefore, the proposed framework should be considered a research-stage approach rather than a clinically validated diagnostic or screening system.

## 7. Conclusions

This study presents an interpretable and computationally efficient framework for ECG rhythm classification based on statistical RR interval variability features. The proposed framework achieved 93% accuracy in the same-dataset ECG-SDS evaluation and 78.6% accuracy in the cross-dataset evaluation, where the model was trained on PhysioNet/CinC 2017 [28] and tested on the synthetic ECG-SDS dataset [20]. The lower performance observed in the cross-dataset setting may reflect dataset shift as well as differences in model configuration and evaluation protocol. The exploratory same-dataset multi-class evaluation on ECG-SDS achieved an accuracy of 91.67% and should be interpreted cautiously because ECG-SDS consists of synthetic ECG recordings, and the multi-class analysis was restricted to its dataset-specific rhythm definitions.

The feature ablation analysis showed that the selected RR interval descriptors collectively provide useful information for classification, while mean RR and heart rate should be regarded as redundant because heart rate was derived directly from mean RR. The grouped ablation further showed that simultaneous removal of mean RR and heart rate reduced the mean classification accuracy from 91.91% to 84.64%, indicating that information related to the average RR interval remains important despite this redundancy. Among the evaluated classifiers, Random Forest offered a favourable balance between accuracy, interpretability, and computational efficiency. The feature-level sensitivity analysis showed relatively stable classification performance under small Gaussian perturbations applied to the standardized extracted features, with performance gradually decreasing as the perturbation magnitude increased. Because these perturbations were applied after RR interval feature extraction rather than to the raw ECG signals, this experiment should be interpreted as a feature-level sensitivity analysis and not as evidence of robustness of the complete ECG-processing pipeline to real-world ECG noise.

Overall, the results demonstrate the potential of statistical RR interval features combined with classical machine learning for compact and interpretable ECG rhythm analysis. However, because ECG-SDS consists of synthetic ECG recordings, the cross-dataset results should be interpreted as a technical assessment of transferability rather than as validation in an independent clinical population. The proposed framework remains at the research stage and requires validation using independent real-world clinical ECG datasets before conclusions regarding clinical generalizability or deployment can be drawn. Future work will focus on evaluation using larger and more diverse real-world clinical ECG datasets, incorporating clinically verified rhythm classes, exploring frequency-domain and nonlinear HRV features, and investigating domain-adaptation techniques to improve cross-dataset generalization while preserving interpretability and computational efficiency.

## Figures and Tables

**Table 1:** Cross-dataset ECG rhythm classification performance on the ECG-SDS dataset.

Class	Precision	Recall	F1-Score	Support
Normal sinus rhythm	0.882	0.804	0.841	1058
Abnormal cardiac rhythm	0.613	0.742	0.671	442
Accuracy	–	–	0.786	1500
95% Bootstrap CI (Accuracy)	–	–	76.47–80.73%	–
Macro Avg	0.747	0.773	0.756	1500
Weighted Avg	0.803	0.786	0.791	1500

**Table 2:** Intra-dataset ECG-SDS classification results using statistical RR interval features.

Class	Precision	Recall	F1-Score	Support
Normal sinus rhythm	0.92	0.99	0.95	317
Abnormal cardiac rhythm	0.97	0.78	0.87	133
Accuracy	–	–	0.93	450
Macro average	0.94	0.89	0.91	450
Weighted average	0.93	0.93	0.93	450

**Table 3:** Performance of the Random Forest classifier for the exploratory three-class same-dataset ECG rhythm classification.

Class	Precision	Recall	F1-Score	Support
Normal	0.91	1.00	0.95	211
Sinus Tachy–Brady	0.92	0.49	0.64	45
AFib	0.93	0.98	0.96	44
Macro average	0.92	0.82	0.85	300
Weighted average	0.92	0.92	0.91	300
**Overall Accuracy**			**91.67% (275/300)**	

**Table 4:** Binary classification accuracy under Gaussian perturbations applied in the standardized feature space.

Noise Level (σ)	Accuracy (%)
0.00	92.89
0.02	93.11
0.05	92.67
0.10	91.78
0.20	90.67

**Table 5:** Feature ablation analysis using repeated stratified train–test splits. Results are reported as mean accuracy ± standard deviation across 10 independent splits.

Feature Configuration	Accuracy
All features	0.9191±0.0105
Without mean RR	0.9193±0.0108
Without SDNN	0.9193±0.0102
Without RMSSD	0.9176±0.0098
Without pNN50	0.9191±0.0095
Without HR	0.9198±0.0104
Without mean RR and HR	0.8464±0.0139

**Table 6:** Contextual comparison of the proposed method with representative ECG arrhythmia classification studies using different datasets and evaluation settings. The reported accuracies are provided for contextual reference and are not intended as direct like-for-like comparisons because the studies differ in datasets, class definitions, features, classifiers, and evaluation protocols.

Reference	Dataset	Features Used	Classifier	Evaluation	Accuracy (%)
Devadas et al., [24]	UCI arrhythmia	Morphological and statistical	SVM/KNN/NB	Intra-dataset	71.4/70.3/62.6
Tunc and Güngör, [25]	UCI arrhythmia	Selected ECG features	KNN/DT/SVM	Intra-dataset	69.66/73.50/81.27
Aziz and Ahmed, [26]	SPH	Raw ECG	CNN	Intra-dataset	68.0
Cai and Zhou, [27]	PhysioNet-derived	Time-domain features	Classical ML	Intra-dataset	75.6
Proposed Method (Binary-Class Classification)	ECG-SDS	RR interval statistics	Random Forest	Intra-dataset	93.0
Proposed Method (Multi-Class Classification)	ECG-SDS	RR interval statistics	Random Forest	Intra-dataset	91.67
Proposed Method (Binary-Class)	PhysioNet and ECG-SDS	RR interval statistics	Random Forest	Cross-dataset	78.6

**Table 7:** Random Forest feature importance analysis.

Feature	Importance
RMSSD	0.312
SDNN	0.248
HR	0.168
MeanRR	0.150
pNN50	0.121

**Table 8:** Comparison of machine learning classifiers using the proposed RR interval feature set under the same 70:30 stratified train–test partition and preprocessing protocol.

Classifier	Accuracy	Precision	Recall	F1-Score
Random Forest	0.9289	0.9720	0.7820	0.8667
SVM	0.9111	1.0000	0.6992	0.8230
KNN	0.9133	0.9519	0.7444	0.8354
Logistic Regression	0.9067	1.0000	0.6842	0.8125

## Data Availability

The dataset analyzed in this study is the ECG-SDS Starter Dataset v1.0 (ecg_sds_starter), acquired from QInsight Labs Pvt. Ltd. (accessed on 20 February 2026 via official distribution). The dataset comprises 1500 synthetic single-lead (Lead II) ECG recordings sampled at 250 Hz across three rhythm classes (normal_sinus, afib_basic, and sinus_tachy_brady). Technical validation and morphological consistency were confirmed by the provider through signal statistical analysis and machine learning benchmark classification (test accuracy: 0.83), as documented in the dataset validation report (docs/validation_report.pdf). As the dataset is entirely synthetic, no human participants were involved, ethical committee approval and patient consent were not required, and all associated metadata are fully simulated and anonymized. The dataset was used in strict compliance with the ECG-SDS Starter Dataset License Agreement (Version 1.0) for non-commercial academic research. Citation: QInsight Labs–ECG-SDS Starter Dataset v1.0. The PhysioNet/Computing in Cardiology Challenge 2017 dataset (Version 1.0.0) used for model development is publicly available through PhysioNet at https://physionet.org/content/challenge-2017/1.0.0/ (accessed on 20 Febuary 2026).
